# Antioxidant capacity, phytochemical profiles, and phenolic metabolomics of selected edible seeds and their sprouts

**DOI:** 10.3389/fnut.2022.1067597

**Published:** 2022-12-14

**Authors:** Hong-Yan Liu, Yi Liu, Ming-Yue Li, Ying-Ying Ge, Fang Geng, Xiao-Qin He, Yu Xia, Bo-Li Guo, Ren-You Gan

**Affiliations:** ^1^Chengdu National Agricultural Science and Technology Center, Research Center for Plants and Human Health, Institute of Urban Agriculture, Chinese Academy of Agricultural Sciences, Chengdu, China; ^2^Key Laboratory of Coarse Cereal Processing (Ministry of Agriculture and Rural Affairs), Sichuan Engineering and Technology Research Center of Coarse Cereal Industrialization, College of Food and Biological Engineering, Chengdu University, Chengdu, China; ^3^Department of Food Science and Technology, School of Agriculture and Biology, Shanghai Jiao Tong University, Shanghai, China; ^4^Institute of Food Science and Technology, Chinese Academy of Agricultural Sciences, Beijing, China

**Keywords:** germination, phenolic, antioxidant activity, sprouts, metabolomics

## Abstract

Sprouts are recognized as nutritional and functional vegetables. In this study, 17 selected seeds were germinated simultaneously. The antioxidant capacity and total phenolic content (TPC) were determined for seeds and sprouts of all species. Both seed and sprout of white radish, with the highest antioxidant capacity, and TPC among all the 17 species, were further determined for phenolic metabolomics. Four phenolic classes with 316 phenolic metabolites were identified. 198 significantly different metabolites with 146 up-regulated and 52 down-regulated were confirmed, and high amounts of phenolic acids and flavonoids were found to be accumulated in the sprout. Several metabolism and biosynthesis, including phenylpropanoid, favone and flavonol, phenylalanine, and various secondary metabolites, were significantly activated. Significant correlations were found among FRAP, DPPH, ABTS, TPC, and phenolic profiles. Therefore, white radish sprout could be served as antioxidant and could be a good source of dietary polyphenols.

## 1 Introduction

Phenolic compounds are secondary metabolites in plants (1). In recent years, phenolic compounds have attracted increasing attention based on their nutrition and health benefit. It is reported that phenolic compounds have protective effect against oxidative reactions, which was connected with diminishing the dangers of cancer, coronary illness and diabetes, restraint of bacterial, inflammation and allergies (2–4).

The edible seeds have been widely reported to show various health effects (5, 6). Germination is a kind of processing method, the germinated sprouts generally accumulated more phenolic compounds which could prevent lipids from oxidation (7, 8), making them as valuable sources of food ingredients. In comparison with seed, germination has many advantages for improving the nutritional and functional qualities, such as the accumulation of many biologically active compounds, including γ-aminobutyric acid, minerals, phenolic acids, flavonoids, vitamins, etc. (9, 10).

At present, the phenolic content as well as the antioxidant activities of many edible seeds and sprouts (e.g., legumes, Brassica, and Gramineae) has been studied. However, the germination condition, and applied antioxidants assay in most previous reports were not uniform, and the differences in growth conditions (light sources and time, different treatment of seeds, and environmental shocks) might lead to dramatic differences in the chemical compounds, metabolomics and antioxidant activity (11–14), making it difficult to compare the results and achieve consistent conclusion directly. In addition, little information was available about the main phenolic compounds in sprout that responsible for these antioxidant properties. As a result, it is necessary to study on phenolic compounds and antioxidant capacity of both seed and sprout in the same germinating condition. Meanwhile, the main metabolites that contribute to the antioxidant activity in seed and sprout must be excavated.

It is not clear how the phenolic compounds and their antioxidant activities change throughout seed germination, and it is necessary to identify the optimum plant species with optimum physiological stages in order to accumulate the maximum phenolic accumulation. As a result, we hypothesized that the phenolic metabolites and their antioxidant activities of sprouts would change with germination days. With the improvement of analytical methods and instrument sensitivity, phenolic metabolomics analysis covering more than 2,000 metabolites could be determined simultaneously. In this study, the total phenolic contents (TPCs) as well as the antioxidant activities of 17 types of seeds and their 6-day sprouts were analyzed. Furthermore, the white radish (with the highest antioxidant capacity and TPC values) was selected for another germination trial in order to investigate the dynamic changes of antioxidant capacity and TPC at different germination stages (1, 2, 3, 4, 5, 6 days, respectively). In addition, the phenolic metabolomics in the white radish seed and sprout extract were analyzed qualitatively and quantitatively by ultra-performance liquid chromatography-electrospray ionization mass spectrometry/mass spectrometry (UPLC-ESI-MS/MS), in order to confirm the main different phenolic compounds after germination. Finally, the relationships between the antioxidant capacity and phenolic profiles were analyzed. The obtained results may provide references for the selection of edible seeds and sprouts with high antioxidant capacity as raw materials for functional foods.

## 2 Materials and methods

### 2.1 Chemicals and reagents

The chemicals of 6-hydroxy-2,5,7,8-tetramethylchromane-2-carboxylic acid (Trolox), 2,2’-azino-bis (3-ethylbenz-thiazoline-6-sulfonic acid) (ABTS), 1,1-diphenyl-2-picryl-hydrazyl (DPPH), 2,4,6-tri(2-pyridyl)-S-triazine (TPTZ), Folin–Ciocalteu’s phenol reagent, and gallic acid and catechin were purchased from Sigma-Aldrich (St. Louis, MO, USA). The sodium acetate, ferric chloride, and Na_2_CO_3_ (> 99%) were purchased from Beijing Solarbio Technology Co., Ltd. (Beijing, China). The methanol (chromatographic grade grade) was obtained from Beijing Yishan Huitong Technology Co., Ltd. (Beijing, China). Milli-Q ultra-pure water was used for all experiments.

### 2.2 Sprout growth and pretreatments

Based on the common grains and vegetables, totally 17 crop seeds of broccoli (*Brassica oleracea* L. var. italic), pakchoi (*Brassica campestris* L. ssp. *chinesis* var. communis), pakchoi seedlings (*Brassica campestris* L. ssp. *chinensis* var. communis), purple radish (*Raphanus sativus* L.), white radish (*Raphanus sativus* L.), sunflower (*Helianthus annuus* L.), water spinach (*Ipomoea aquatica* Forssk.), barley (*Hordeum vulgare* L.), triticale (*X Triticosecale* Wittm.), wheat (*Triticum aestivum* L.), perilla (*Perilla frutesches* L. Britt), alfalfa (*Medicago sativa* L.), lentil (*Lens culinaris* Medik.), pea (*Pisum sativum* L.), pine willow (*Lathyrus quinquenervius* (Miq.) Litv), okra (*Abelmoschus esculentus* L. Moench), and vanilla (*Vanilla planifolia* L.) were purchased from Weifang Shuishengtian Agriculture Technology Co., Ltd. (Weifang, China). All the seeds were collected at the same region in the year of 2021.

After cleaning and picking out bad seeds, the seeds were soaking for 5–24 h, and germinated for 6 days with 12 h daylight and 12 h darkness each day. The germination temperature was 25°C and the humidity was kept at 90%. The de-ionized water was used as substrate to germinate, and all the sprouts were sampled after 6 days germination. Furthermore, the white radish sprouts (with the highest antioxidant capacity and TPC) were sampled after 24, 48, 72, 96, and 120 h of germination with the same seeds. After harvest, the sprout samples were frozen with liquid nitrogen treatment immediately, and then freeze-dried for 24 h. Finally, seeds and sprouts were further milled in a miller (Tube Mill 100 control, IKA, Germany) to obtain powder samples. The growth status of sprouts was shown in Supplementary Figure 1.

### 2.3 Analysis of the total phenolic content

About 0.5 g of seed or sprout powder by adding 10 mL of 80% (v/v) ethanol was shaken for 24 h at 22^°^C, the solution was then centrifuged with 3,000 × g for 30 min at 4^°^C, the supernatant was collected and diluted by 80% of ethanol before determination the measurement. All the contents were finally corrected with the dilution fold finally. The TPC was detected by Folin–Ciocalteu method described previously with a little modification (15), and the absorbance at 760 nm was determined. Finally, the TPC of seed or sprout was expressed as mg gallic acid equivalent (GAE)/100 g DW. Each sample was determined in triplicate.

### 2.4 Analysis of the antioxidant activities

The sample extraction and dilution methods were the same as the methods for analysis of TPC, all the values were finally corrected with the dilution fold, and each sample was determined in triplicate.

The DPPH working solution was prepared by adjusting the DPPH solution (100 μM) with 80% methanol to an absorbance of 0.70 ± 0.05 at 515 nm. 100 μL of the properly diluted supernatant with 3.9 mL working solution was mixed to react for 60 min in dark at room temperature, and the solution was then detected at 515 nm. Finally, μmol of Trolox/g of seeds and sprouts were used to express DPPH values.

A total of 100 μL of the properly diluted supernatant was mixed with 3 mL FRAP working solution (300 mmol/L of sodium acetate buffer, 10 mmol/L of TPTZ solution, and 20 mmol/L of ferric chloride solution, 10:1:1 [v/v/v)] for 4 min at room temperature, and the absorbance at 593 nm was tested further. Finally, the FRAP value of seed and sprout was shown as μmol Fe(II)/g DW.

The 7 mmol/L ABTS solution and 2.45 mmol/L potassium persulfate solution were mixed at a volume ratio of 1:1 to obtain the ABTS stock solution. The ABTS stock solution was diluted with ultra-pure water to ensure its absorbance was 0.710 ± 0.05 at 734 nm as ABTS working solution. When determination, 100 μL of the properly diluted supernatant mixed with 3.9 mL working solution was reacted at room temperature for 6 min in darkness. The absorbance of the solution at 734 nm was then detected. Finally, the ABTS value of seed or sprout was expressed as μmol Trolox/g dry weight (DW).

### 2.5 Metabolomics analysis

White radish seed and sprout samples were freeze-dried by a vacuum freeze-dryer (SJIA-5S, Ningbo Shuangjia Instrument Co., Ltd., Ningbo, China), and crushed by a mixer mill (MM 400, Retsch) as fine powder. The prepared powder sample (50 mg) was then dissolved by 1.2 mL 70% methanol solution, and vortex for 30 s every 30 min with six times in total. Finally, the extracts were centrifuged for 3 min at 12,000 rpm, and then filtrated with 0.22 μm filter.

The extracts were determined by UPLC-ESI-MS/MS system (UPLC, SHIMADZU Nexera X2HPLC-MS/MS system; MS, Applied Biosystems 4500 Q TRAP). The HPLC column was SB-C18 (2.1 mm × 100 mm, 1.8 μm). The solvent system was composed with water containing 0.1% formic acid (solvent A) and 0.1% (v/v) formic acid in acetonitrile (solvent B). The program conditions were set as follows: 5–95% B from 0 to 9 min and kept for 1 min, and 95–5% B from 10 to 11 min and kept for 3 min. The temperature, low velocity, and injection volume were 40°C, 0.35 mL/min, and 4 μL, respectively.

The effluent was alternatively connected to an ESI-triple quadrupole-linear ion trap (QTRAP)-MS, equipped with an ESI Turbo Ion-Spray interface, operating in positive and negative ion modes and controlled by the Analyst 1.6.3 software (AB Sciex, Framingham, MA, USA). In triple quadrupole (QqQ) and Linear ion trap (LIT) modes, 10 and 100 μmol/L polypropylene glycol solutions were used for instrument tuning and quality calibration, respectively, QqQ scans were acquired as multiple reaction monitoring mode (MRM) experiments with collision gas (nitrogen) set to medium, declustering potential (DP) and collision energy (CE) for individual MRM transitions were done with further DP and CE optimization. The ESI source was operated as follows: temperature 550°C. The ion spray voltage in positive and negative mode, respectively, was 5.5 and 4.5 kV. Set at 50, 60, and 25 psi for the ion source gases I, II, and curtain gas, respectively. A pooled sample was run after every two samples to serve as quality control (QC) to estimate the variables.

Phenolic compounds were tentatively identified according to a previous study (16). Both primary and secondary MS information was compared with the database self-builted by Metware Biotechnology Co., Ltd. (Wuhan, China) as well as the publicly available databases covering metabolites. In order to identify the molecules qualitatively, metabolite primary and secondary mass spectrometry data, including the accurate precursor ion (Q1), and product ion (Q3) value, retention times (RT), declustering potential (DP), and collision energy (CE) were subjected to qualitative analysis by referencing self-built database MWDB (Metware Biotechnology Co., Ltd., Wuhan, China) and public mass spectrometry databases such as MassBank,^1^ KNAPSAcK,^2^ HMDB,^3^ and METLIN,^4^ these information in the samples were intelligently matched with the database one by one, and the mass tolerance of MS and MS/MS were set as ± 10 ppm.

Triple quadrupole mass spectrometry was responsible for the quantification of metabolites. Based on MRM scanning, the quadrupole first searched for precursor ions (parent ions) of target substances while screening any ions derived from substances of different molecular weights to eliminate their interference preliminarily. The precursor ions were fragmented *via* induced ionization in the collision chamber to form many fragment ions, which were then filtered through QqQs to select single-fragment ions with the desired characteristics. MultiQuant quantitative software was used to integrate and to obtain quantitative data. After the metabolite mass spectrometry data were obtained for each sample, all the mass spectrum peaks were subjected to area integration. In order to qualitative and quantitative analyze of each metabolite in different samples more accurately, each mass spectrum peak was corrected according to the RT and peak shape of each metabolite. The relative content of each metabolite was represented with chromatographic peak area integrals.

### 2.6 Statistical analysis

The results were presented as mean ± standard deviation (SD). The ANOVA and Pearson correlation analysis were analyzed by SPSS statistical software (IBM SPSS Statistics 20.0, SPSS Inc., Chicago, IL). PCA (principal component analysis) and OPLS-DA (orthogonal partial least squares-discrimination analysis) were conducted by R.^5^ The metabolism data was normalized by unit variance scaling and zero-centered to ensure that the processed data conformed to standard normal distribution.

## 3 Results

### 3.1 The TPC and antioxidant activity of seeds and sprouts

**FIGURE 1:**
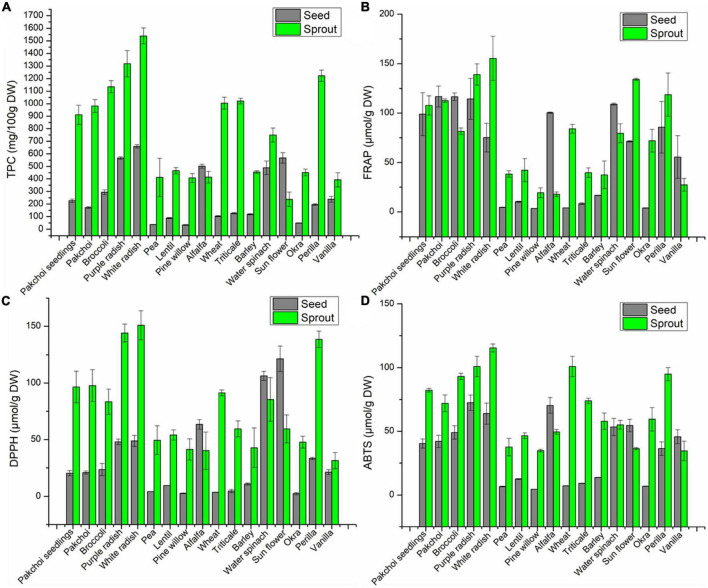
The TPC **(A)**, FRAP **(B)**, DPPH **(C),** and ABTS **(D)** values of 17 seeds and their sprouts.

The TPC of all sprouts were higher than corresponding seeds, except for alfalfa and oil sunflower (Figure 1A). What’s more, the TPC of white radish sprout and its seed were highest among all the sprouts or seeds, respectively, reaching 15.39 and 6.60 mg GAE/g DW, followed by purple radish with 13.19 and 5.67 mg GAE/g DW for sprout and seed, respectively. The TPC accumulated within 6 days of germination accounted for 4.03-fold (pakchoi seedings), 5.71-fold (pakchoi), 3.86-fold (broccoli), 2.32-fold (purple radish), 2.33-fold (white radish), 11.20-fold (pine willow), 0.82-fold (alfalfa), 9.73-fold (wheat), 8.05-fold (Triticale), 3.84-fold (barely), 1.53-fold (water spinach), 0.42-fold (sunflower), 9.23-fold (okra), 6.23-fold (Perilla), and 1.65-fold (vanilla) compared with their seeds.

The results of antioxidant activity based on the FRAP assay showed that among 17 edible seeds and sprouts, the antioxidant activities in most of the sprouts were higher than those of their seeds, which was inconsistent or contrary to the TPC results among these nine seeds (Figure 1B). The results of antioxidant activity measured based on DPPH assay showed that except for spinach, alfalfa and oil sunflower, the antioxidant activities of the remaining 14 sprouts were higher than those of their seeds (Figure 1C). The results of antioxidant activity based on the ABTS assay showed that except for alfalfa and oil sunflower, the antioxidant activities of the remaining 15 sprouts were higher than those of their seeds (Figure 1D).

In addition, the highest antioxidant activity in all sprout was found in white radish, the FRAP, DPPH, and ABTS values, respectively, were 155.4 μmol Fe(II)/g DW, 150.9 μmol Trolox/g DW, and 115.4 μmol Trolox/g DW. Followed by purple radish, respectively were 139.0 μmol Fe (II)/g DW, 144.1 μmol Trolox/g DW, and 101.0 μmol Trolox/g DW.

### 3.2 The dynamic changes of TPC and antioxidant activity during germination

**FIGURE 2:**
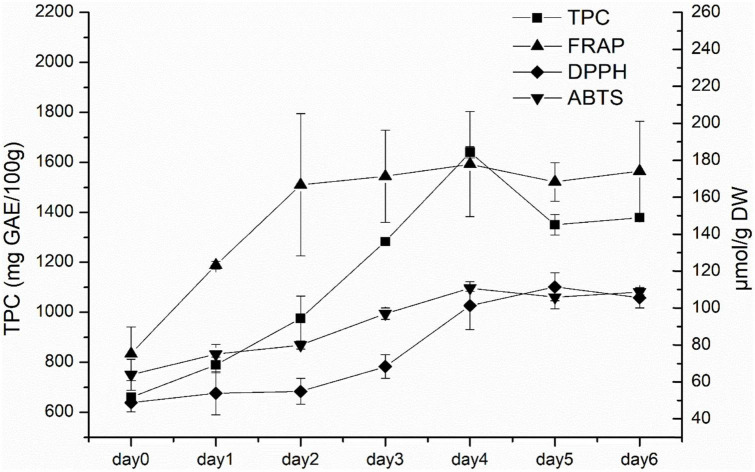
The TPC and antioxidant capacities (ABTS, DPPH, and FRAP) of white radish during germination.

According to the above experimental results, white radish sprouts showed the highest antioxidant capacities. As a result, they were selected for subsequent research. The dynamic changes of TPC, FRAP, DPPH, and ABTS in white radish during germination were analyzed (Figure 2). TPC generally showed an increasing trend in the first 4 days, and then decreased afterward. The FRAP value increased rapidly in the first 3 days and kept stable in the following germination days. The DPPH value increased with the germination time during the first 5 days and decreased slightly on the sixth day. The ABTS value generally increased during the first 4 days, this was consistent with the TPC result.

### 3.3 Metabolomics analysis of white radish seed and its sprouts

#### 3.3.1 The differences in phenolic classes between seed and sprout

**FIGURE 3:**
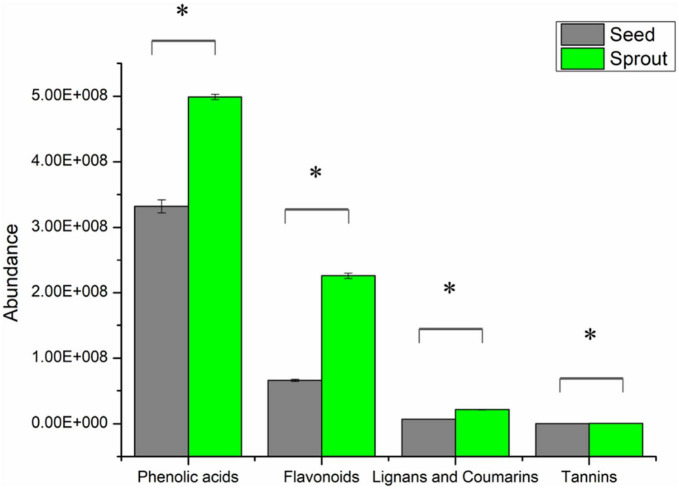
The abundance of phenolic classes between white radish seed and sprout. *Indicated significantly different (*P* < 0.05).

In total, 316 phenolic metabolites were identified, including 155 phenolic acids, 129 flavanols, 29 lignans, and coumarins, and 3 tannins. After comparing the sum of each phenolic class between seed and sprout (Supplementary Table 1), the abundance of each phenolic class in radish sprout was significantly higher than those in seed by *t*-test (*P* < 0.05) (Figure 3).

The proportion of QC samples with CV values less than 0.3 exceeds 75%, indicating that the stability of this experimental data (Supplementary Figure 2A). The TIC plots for three QC samples were overlapped both in positive and negative mode (Supplementary Figures 2B,C), indicating this method was extremely stable and reliable.

#### 3.3.2 The differences in phenolic compounds between seed and sprout

According to the PCA result, the first three principal components were extracted to be 84.47, 5.08, and 4.04%, respectively (Supplementary Figure 3). These plots showed that the seed and sprout of white radish could be clearly separated by phenolic compounds. These results suggested significant differences in the phenolic profiles of white radish during germination.

**FIGURE 4:**
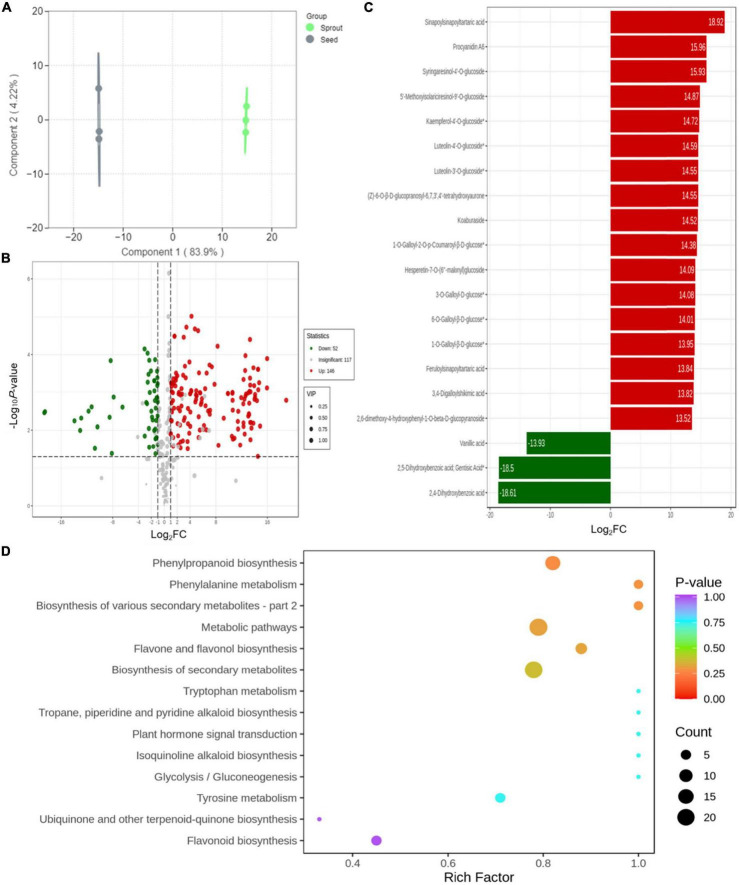
**(A)** Orthogonal partial least squares-discrimination analysis result for discriminating of white radish seed and sprout; **(B)** volcano plots of the up- and down-accumulated metabolites for comparison groups of seed and sprout; **(C)** bar chart of the top 20 differentially abundant phenolic compounds with the largest fold change (FC) value; **(D)** KEGG enrichment of differential metabolites in the comparison of seed and sprout.

Orthogonal partial least squares-discrimination analysis was employed to investigate the different compounds between radish seed and sprout (Figure 4A). Differential metabolites were determined by VIP (VIP ≥ 1) and absolute Log_2_FC (| Log_2_FC| ≥ 1.0). VIP values were extracted from OPLS-DA result, which also contain score plots and permutation plots, was generated using R package MetaboAnalyst R. The data was log transform (log_2_) and mean centering before OPLS-DA. In order to avoid overfitting, a permutation test (200 permutations) was performed. The validation result of the OPLS-DA model was *R*^2^X = 0.881, *R*^2^Y = 1, *Q*^2^ = 0.998. The *R*^2^Y and *Q*^2^-values exceed 0.9, indicating that the model was excellent, with good predictability and high fit accuracy (Supplementary Figure 4). There were 251 phenolic compounds whose VIP scores were higher than 1. Based on *P* < 0.05 and VIP ≥ 1, totally 198 significantly different metabolites were observed between white radish seed and sprout, with 52 down-regulated and 146 up-regulated (Figure 4B). In particular, there were 34 down-regulated and 72 up-regulated in phenolic acids, 14 down-regulated and 58 up-regulated in flavonoids, 3 down-regulated and 19 up-regulated in lignans and coumarins, 2 down-regulated and 1 up-regulated in tannins. The screening of differentially abundant phenolic metabolites between white radish seed and sprout was based on FC > 2 (or < 0.5) and *P* < 0.05, compounds with top 20 fold change were listed in Figure 4B. There were 17 compounds (including sinapoylsinapoyltartaric acid, procyanidin A6, syringaresinol-4’-O-glucoside, etc.) were up-regulated (red) and 3 compounds (vanillic acid, 2, 4-dihydroxybenzoic acid, and 2, 5-dihydroxybenzoic acid) were down-regulated (green) (Figure 4C).

The metabolite accumulation information was studied by the public databases of Kyoto Encyclopedia of Genes and Genomes (KEGG) in this work. The differential metabolites for white radish seed vs. sprout were involved in 14 pathways presented in bubble plots. Several metabolic pathways including “phenylpropanoid biosynthesis,” “phenylalanine metabolism,” “biosynthesis of various secondary metabolites,” “biosynthesis of secondary metabolites,” “metabolic pathways,” and “flavone and flavonol biosynthesis” were significantly enriched (*P* < 0.05) (Figure 4D).

**FIGURE 5:**
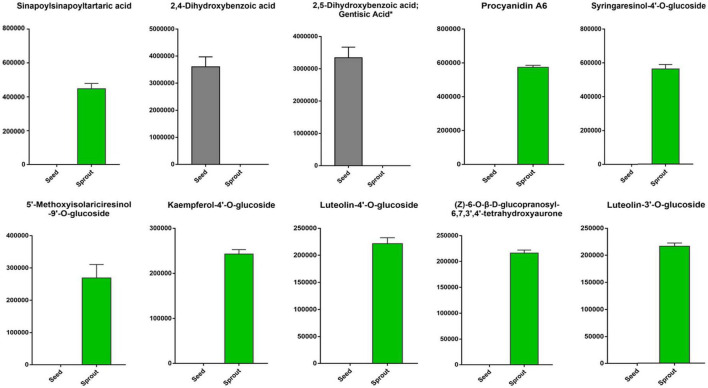
The top 10 differentially abundant phenolic compounds with the largest fold change (FC) value in the pairwise comparison of white radish seed and sprout.

The top 10 differentially abundant phenolic compounds with the largest fold change (FC) value between white radish seed and sprout were listed in Figure 5. Among them, 2,5-dihydroxybenzoic acid (gentisic acid) and 2,4-dihydroxybenzoic acid were not identified in white radish sprout, but were identified in seeds. The other six phenolic compounds, including sinapoylsinapoyltartaric acid, procyanidin A6, syringaresinol-4′-O-glucoside, 5′-methoxyisolariciresinol-9′-O-glucoside, kaempferol-4′-O-glucoside, luteolin-4′-O-glucoside, (Z)-6-O-β-D-glucopranosyl-6,7,3′,4′-tetrahydroxyaurone, and luteolin-3′-O-glucoside were only found in white radish sprouts.

#### 3.3.3 Correlation analysis between antioxidant activity and main phenolic profiles

**FIGURE 6:**
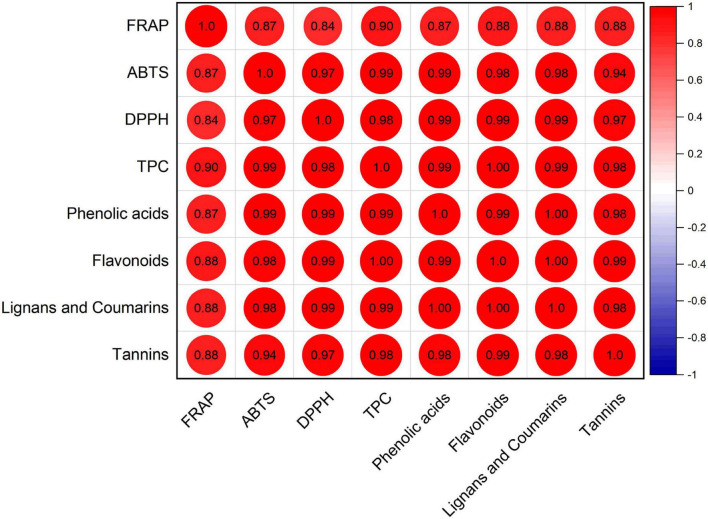
Correlation analysis among antioxidant capacity, TPC, and phenolic profiles.

Furthermore, Pearson correlation analysis was conducted between FRAP, DPPH, ABTS, TPC, and each phenolic class of all white radish seeds and sprouts (Figure 6). Significantly positive correlations were obtained among FRAP, DPPH, ABTS, TPC, phenolic acids, flavonoids, lignans, and coumarins, and tannins (*P* < 0.05). The Pearson correlation coefficients ranged from 0.84 to 0.99, indicating very strong correlation among these parameters.

## 4 Discussion

The enhancement in phenolic content and antioxidant activity after germination has been broadly reported in wheat (17–19), barley (20), and radish (21). The TPC (DW) and DPPH values of wheat, radish, broccoli, lentil, and alfalfa sprouts are significantly higher than the dormant seed and imbibed seed (22). Also, some studies showed that TPC and antioxidant activities significantly increased during germination in lentil (4, 23) and legumes (24). The TPC, DPPH values, and ABTS values of radish and broccoli sprouts after 5-day growth were higher than those in their seeds (25). These results were consistent with ours. Significant changes in phenolic composition and antioxidant activity were found in the 17 edible seeds and sprouts, the reason might be associated with the initiation of endogenous enzymes and complicated biochemical metabolism (17). However, some samples with higher TPC have lower antioxidant capacity, this could be explained by the fact that some other compounds in extracts influence the responses of the FRAP, ABTS, and DPPH reagents, and this similar results was observed in previous studies (15, 25).

The TPC and DPPH values of 6-day-old alfalfa sprouts were lower than their seeds (26), we also found a similar result in the study. However, the TPC, DPPH, and ABTS values of alfalfa and sunflower seeds were higher than those in their sprouts, indicating that germination might cause a slight reduction of phenolics in some cases. Phenolic compounds are easily to lose while soaking of seeds since they are slightly soluble in water. In addition, they may also be utilized as precursors of cell walls, hormones, and other control substances during growing (19, 27).

In a previous study, the TPC and ABTS of radish both in 5-day old sprouts were higher than those in broccoli and sunflower sprouts (25), The TPC (dry mass) in radish seed was higher than those in broccoli, followed by sunflower, sunflower, alfalfa, and wheat (22, 25). The DPPH, FRAP, and ABTS of lentil seed were higher than those in pea (white) (28). Meanwhile, the TPC and antioxidant capacity of barely were reported to be higher than those in wheat or rye (29), these results were consistent with ours. We also found that the TPC and antioxidant capacity of radish were highest among all the samples, this indicated that the white radish accumulated more phenolic contents than other kinds of seeds during germination, probably due to the higher activity of enzymes for phenolic synthesis in the radish sprout (3).

According to the result of phenolic metabolomics, all four classes of phenolic profiles were significant differences between seed and sprout, and most phenolic compounds were significantly enhanced after germination. Phenolic acids (both free and bound) and flavonoids were the main phenolic profiles in radish seed and sprout, which was consistent with previous results (25). By comparing the top 20 compounds with the largest VIP values in the OPLS-DA model, and the top 20 phenolic compounds with the largest FC, the procyanidin A6 (belong to tannins), glucose (belong to phenolic acids), luteolin (belong to flavonoids) and glucoside (belong to flavonoids) were the most different compounds. According to a previous study, the phenolics, ascorbic acid, flavonoids, and glucosinolates were the major compounds in *Brassicaceae* sprouts such as radish (25, 30, 31). Meanwhile, many pathways were significantly activated, including phenylpropanoid, favone, and flavonol, phenylalanine, and various secondary metabolites. Among them, the phenylpropanoid biosynthesis pathway was reported as a key pathway associated with the production of phenolic acids, lignins, tannins, etc. (32).

The correlation coefficient between antioxidant activity (FRAP, ABTS, and DPPH) and TPC were 0.90, 0.99, and 0.98 for extracts originated from seeds and sprouts. A previous study showed that the antioxidant activity evaluated by FRAP assays was significantly correlated with the TPC (*r* = 0.773) and bound phenolic acids (*r* = 0.874) (25). Our study also showed that significant correlations were found between antioxidant activity and phenolic classes (phenolic acids, flavonoids, lignans and coumarins, and tannins (*P* < 0.05). The TPC, phenolic acids, flavonoids, lignans and coumarins, and tannins also showed significant positive correlations with antioxidant activity in DPPH and ABTS assays (*P* < 0.05). It was previously known that flavonoids were potent antioxidants. Therefore, increased relative amounts of flavonoids and their aglycon may be associated with increased total antioxidant activity (16, 33). Additionally, phenolic acids, which are commonly present in plant cell walls as an integrated component with polysaccharides, also shown antioxidant activity (34, 35). The glycoside compounds in phenolic acids also could protect the oxidative damage and antioxidant effects (36). The findings above imply that phenolic compounds might be a reliable predictor of *in vitro* antioxidant activity in edible seeds and spouts.

## Data availability statement

The original contributions presented in this study are included in the article/Supplementary material, further inquiries can be directed to the corresponding authors.

## Author contributions

H-YL: conceptualization, methodology, and writing—original draft. YL: data curation. M-YL and X-QH: visualization and resources. Y-YG, FG, and YX: visualization and investigation. B-LG: conceptualization and data curation. R-YG: writing—review and editing, supervision, and funding acquisition. All authors contributed to the article and approved the submitted version.

## Footnotes

1
http://www.massbank.jp

2
http://kanaya.naist.jp/KNApSAcK

3
http://www.hmdb.ca

4
http://metlin.scripps.edu/index.php

5
http://www.r-project.org/
