# Supplementary material for: Antioxidant capacity, phytochemical profiles, and phenolic metabolomics of selected edible seeds and their sprouts
Source: Front Nutr. 2022 Dec 14;9:1067597. doi: 10.3389/fnut.2022.1067597 (PMC9798843; doi:10.3389/fnut.2022.1067597)
Supplement: Supplementary file 1 [file Table_1.docx]

Supplementary Material


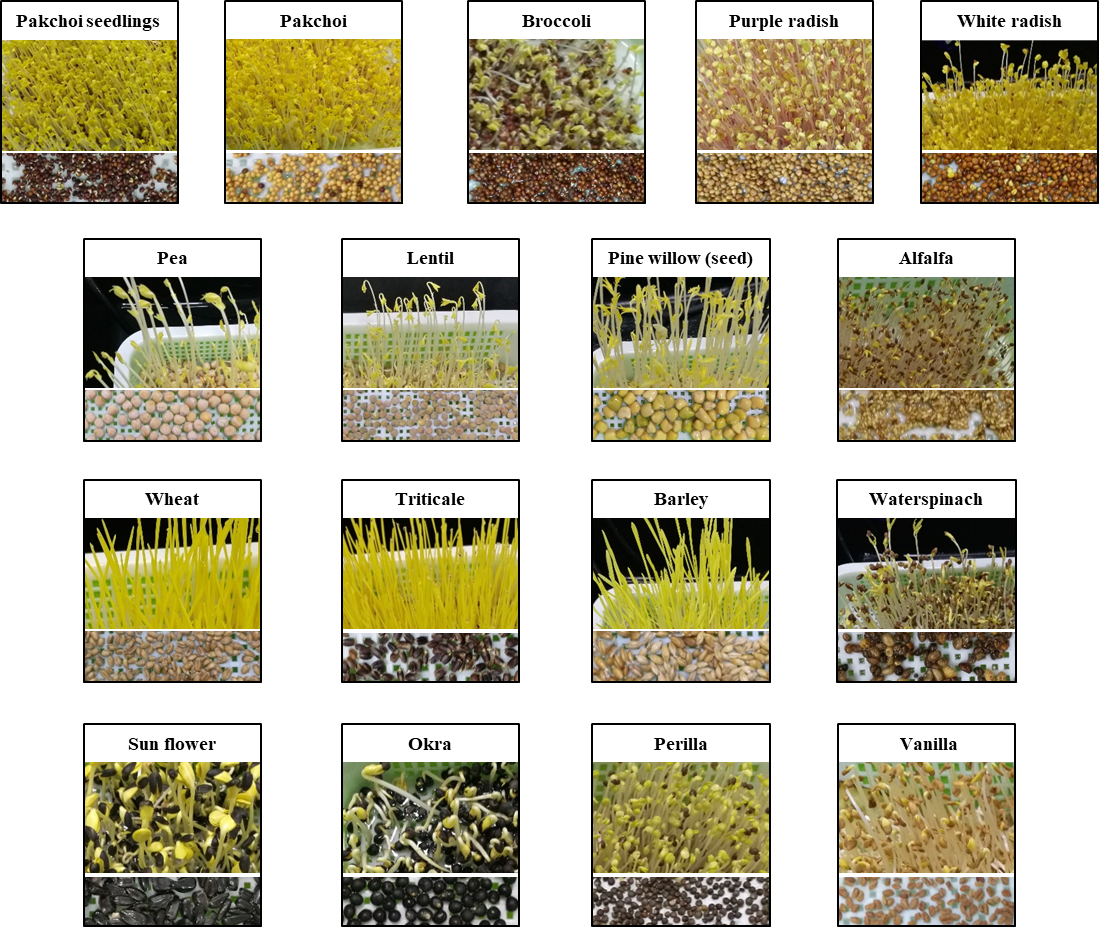


**Supplementary Figure S1.** the status of crop seeds and their sprouts

**
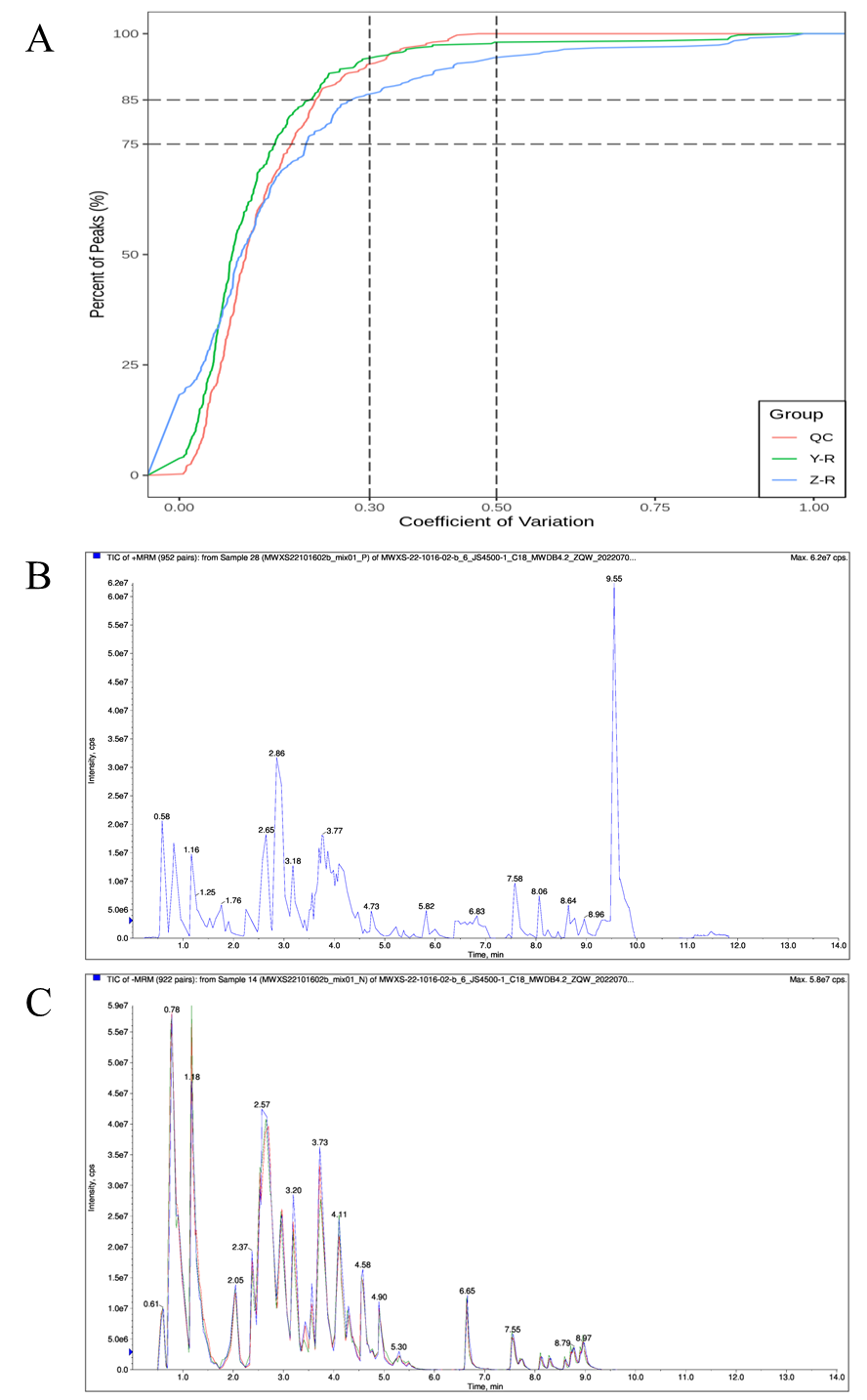
**

**Supplementary Figure S2.** A, the CV distribution map of each group; B, the TIC plot of QC samples in positive mode; C, the TIC plot of QC samples in negative mode


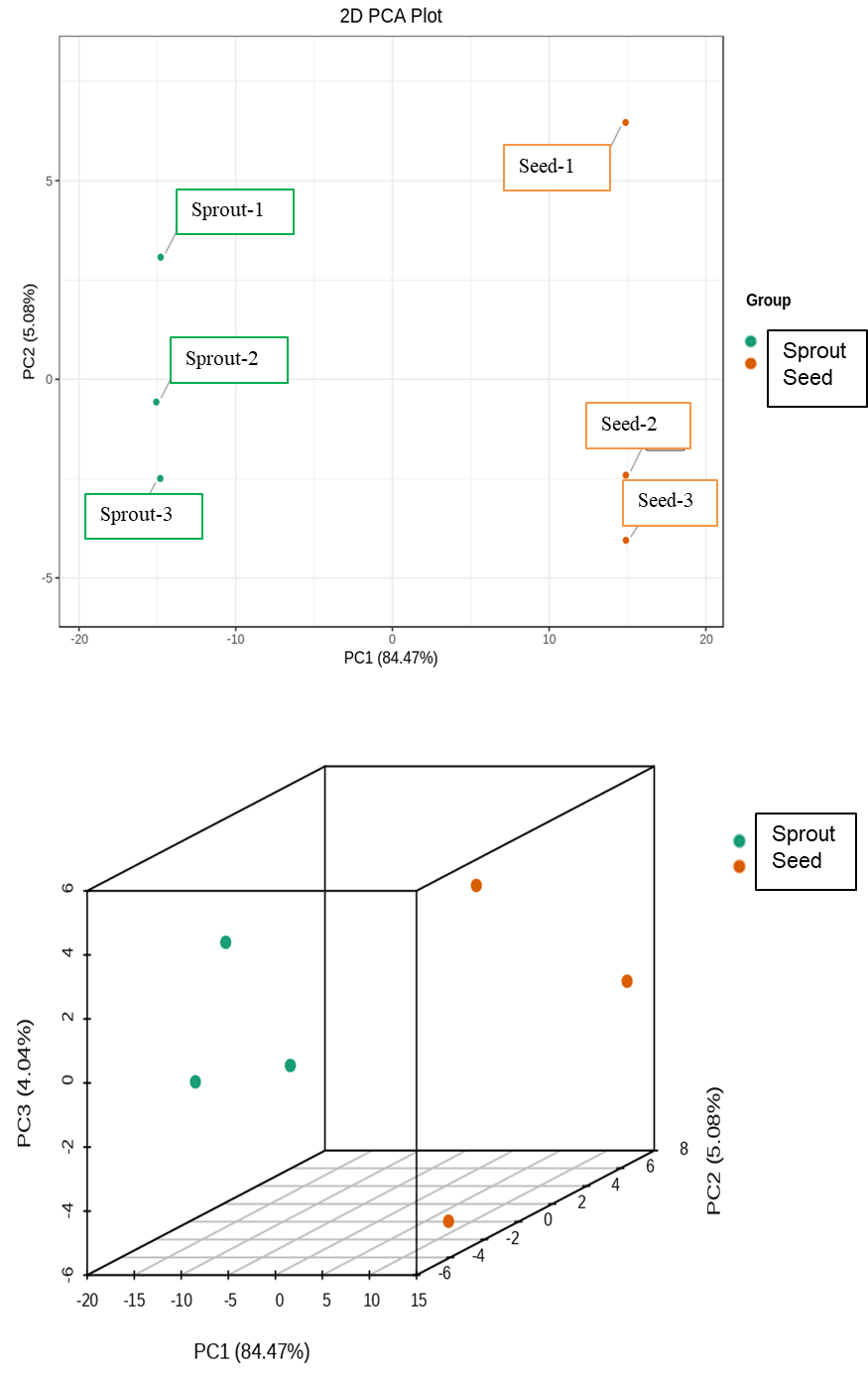


**Supplementary Figure S3.** PCA result of white radish seed and sprout


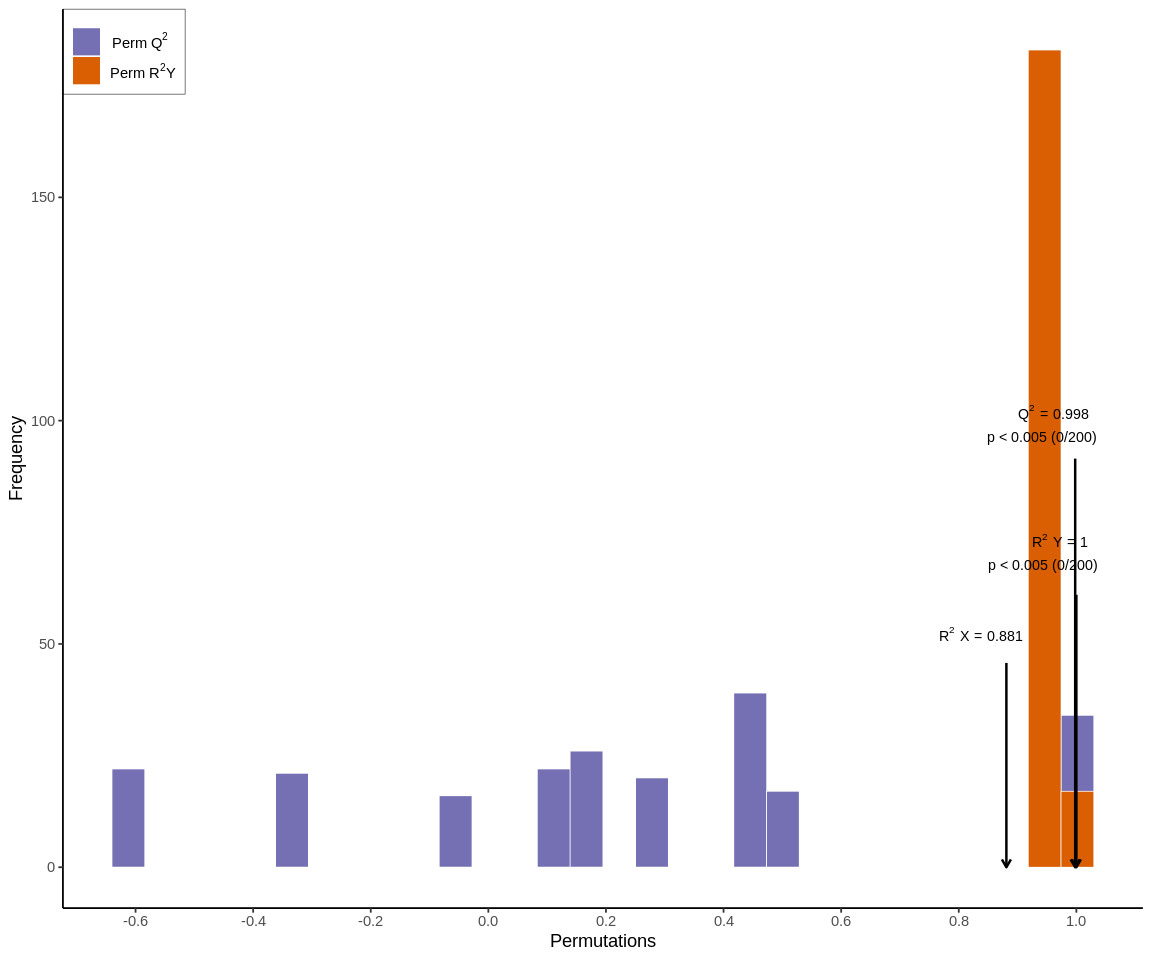


**Supplementary Figure S4.** the OPLS-DA result of white radish seed and sprout
